# BFP Net: Balanced Feature Pyramid Network for Small Apple Detection in Complex Orchard Environment

**DOI:** 10.34133/2022/9892464

**Published:** 2022-09-24

**Authors:** Meili Sun, Liancheng Xu, Xiude Chen, Ze Ji, Yuanjie Zheng, Weikuan Jia

**Affiliations:** ^1^School of Information Science and Engineering, Shandong Normal University, Jinan, China; ^2^Key Laboratory of Facility Agriculture Measurement and Control Technology and Equipment of Machinery Industry, Zhenjiang 212013, China; ^3^National Engineering Research Center for Apple, Shandong Agriculture University, Taian 271018, China; ^4^School of Engineering, Cardiff University, Cardiff CF24 3AA, UK

## Abstract

Despite of significant achievements made in the detection of target fruits, small fruit detection remains a great challenge, especially for immature small green fruits with a few pixels. The closeness of color between the fruit skin and the background greatly increases the difficulty of locating small target fruits in the natural orchard environment. In this paper, we propose a balanced feature pyramid network (BFP Net) for small apple detection. This network can balance information mapped to small apples from two perspectives: multiple-scale fruits on the different layers of FPN and a characteristic of a new extended feature from the output of ResNet50 conv1. Specifically, we design a weight-like feature fusion architecture on the lateral connection and top-down structure to alleviate the small-scale information imbalance on the different layers of FPN. Moreover, a new extended layer from ResNet50 conv1 is embedded into the lowest layer of standard FPN, and a decoupled-aggregated module is devised on this new extended layer of FPN to complement spatial location information and relieve the problem of locating small apple. In addition, a feature Kullback-Leibler distillation loss is introduced to transfer favorable knowledge from the teacher model to the student model. Experimental results show that AP_S_ of our method reaches 47.0%, 42.2%, and 35.6% on the benchmark of the GreenApple, MinneApple, and Pascal VOC, respectively. Overall, our method is not only slightly better than some state-of-the-art methods but also has a good generalization performance.

## 1. Introduction

Fruit detection [1–3], a fundamental task for intelligence agriculture [4, 5], is the basis for the realization of yield prediction [6, 7], pesticide spraying [8], fruit harvesting [9, 10], and automatic monitoring of the whole process of fruit growth [11]. At present, the fruit detection algorithms are mainly based on the general object detection methods, which are optimized to use in the real orchard environment. However, the general object detection algorithms have the characteristic that the precision of small-scale objects is lower than that of large-scale objects. Strictly, the precision of small-scale objects is about half of that of large-scale objects [12], which greatly limits the improvement of fruit detection performance. Therefore, how to improve the precision of fruit detection, especially the precision of small fruits, has attracted the attention of many scholars at home and abroad. Currently, the biggest problem lies with no strict and clear definition of small objects. One definition refers to fruits with smaller physical size in the real world, such as small fruits in the early stages of growth. Another definition is based on the evaluation index of MS COCO [13] and defines the object area with the number of pixels less than 32^2^ as the small objects. More precisely, objects defined by the latter are divided into small objects with 0 < area < 32^2^, medium objects with 32^2^ < area < 96^2^, and large objects with area > 96^2^ according to the number of pixels in the object area. In this paper, the latter is adopted to define the small apples.

Traditional small object detection methods mainly based on the machine learning [14, 15] and digital image processing such as texture information [16] and geometric information [17] were employed to identify and detect the target fruits. With the emergence of graphics processing units (GPUs), a series of small object detection models [18–22] have been proposed. The multiple-scale feature fusion architectures such as feature pyramid network (FPN) [23] and its variants are a typical trick to improve the performance of small object detection. FPN incorporates multiscale features through the lateral connections and a top-down structure. Since the small objects occupying a small number of pixels cannot meet the needs of target detection, the contextual information is integrated into all objects [12], especially small-scale objects. Contextual information about objects can expand the receptive field of objects to complement features of small objects. Although the performance of small targets can be improved to some extent via contextual information, there is still a large gap between the detection precision of small targets and large objects [12]. The small number of pixels occupied by small objects is an important factor hindering the improvement of small object detection performance. Therefore, how to increase the small number of pixels occupied by small objects is still the key to solving the problem. Based on this point of view, a series of small object detection algorithms based on generative adversarial networks (GANs) such as ESRGAN [24] and EESRGAN [25] are derived to generate high-resolution feature mapping and to increase the number of pixels of a small object. An extended feature map from the output of the ResNet/ResNeXt conv1 without the pooling operation was introduced to generate a high-resolution feature map and a foreground-background-balanced loss based on the distillation mechanism was also applied to balance the foreground and background [26].

In this paper, we take Faster R-CNN [27] as the baseline and optimize feature pyramid network to make it suitable for small apple detection. The Faster R-CNN consists of three components: ResNet50 [28], FPN, and a detector. Although FPN can incorporate multiple-scale features, there are unbalanced characteristics of different scale information in each feature layer of FPN. In other words, the fusion feature by element-wise addition of equal proportion will affect the precision of small apple detection. To present this problem more clearly, we quantified the prediction results of different scales of apples on the different resolution feature layer of the FPN structure.

**Figure 1:**
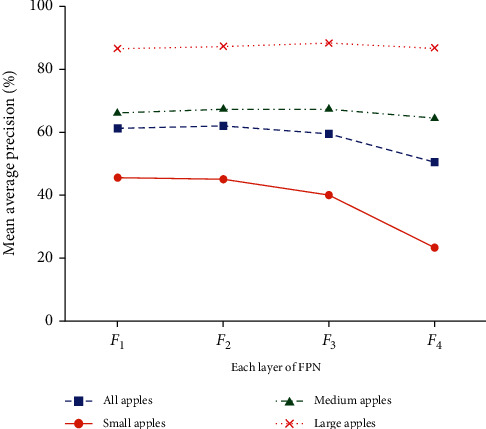
The mean average precision of all apples, small-scale, medium-scale, and large-scale apples on each layer of the standard FPN of Faster R-CNN on the GreenApple.

Figure 1 presents the prediction results of each feature layer of FPN on the Faster R-CNN based on GreenApple, where the horizontal axis represents a certain FPN layer under the standard FPN and the vertical axis represents the mean average precision of the corresponding feature layer. In Figure 1, the precision of small apples falls dramatically with the increase of the number of layers compared with large-scale and medium-scale apples. Despite pyramid shape features being computed in an element-wise addition manner to incorporate multiple-resolution feature maps, the equal proportion addition way does not take into account the characteristics of the features of each layer. The equal proportion feature fusion way degrades the performance of small apple detection. Further, the precision of small apples is poor even on the lowest layer in the standard FPN. The main reason is that the pooling-based downsampling is to filter out the small apples feature information, which leads to fewer features that can be mapped into small apples and makes it difficult to locate small apples.

To alleviate the information imbalance of multiscale objects between each layer of FPN and difficulty of locating small green apples, we propose a balanced feature pyramid network, namely, BFP Net, which can balance information from multiscale apples and the feature characteristic. Specifically, we design a weight-like feature pyramid fusion to balance the small apple information in each feature fusion structure of the vanilla FPN. Meanwhile, a feature of high resolution before pooling in the backbone is first introduced as an extended feature embedded into the lowest layer of FPN. Here, this new feature is different from the extended feature in EFPN [26]. The EFPN extended feature comes from the output of the second stage of ResNet/ResNeXt without pooling layer while the new extended feature is the input of pooling layer. Then, we devise a decoupled-aggregated module with a feature filter to alleviate the problem of difficulty in locating and apply to the new extended layer of FPN. The dual-path decoupled module decouples a feature into a spatial-aware feature and a content-aware feature to relieve the problem of difficulty in locating due to small pixels occupied by small apples and the closeness of color green apples between the fruit skin and the background. Note that the spatial-aware feature is utilized to complement the spatial information into a content-based feature map. An aggregated architecture is applied to incorporate spatial-aware and content-aware information in a point-wise addition manner. One significant advantage of the decoupled-aggregated module is that a feature with high resolution can contain both the semantic information and the location information of the small green apples. To make the consistent distribution of extended layer feature and the lowest feature of standard FPN, a Kullback-Leibler distillation loss is introduced to transfer advantage knowledge from the teacher model to the student model.

We evaluate our method and other state-of-the-art methods on the dataset benchmark of GreenApple and MinneApple, where the performance of each component is evaluated on the GreenApple. The experimental results illustrate that our method is slightly better than other state-of-the-art methods on the GreenApple and MinneApple. In addition, our method has a strong generalization performance on the generic object detection dataset of Pascal VOC.

For clarity, the main contributions of our work can be summarized as follows:

- The balanced feature pyramid network is proposed to improve the performance of small apple detection
- A weight-like feature fusion structure is designed to balance feature information of small apples on different layers of standard FPN
- A decoupled-aggregated module with a feature filter is devised to decouple and aggregate features on the new extended layer differing from the EFPN, where the decoupled structure is applied to remedy difficulty of locating. In addition, a Kullback-Leibler distillation loss is applied to transfer advantage knowledge from the teacher model to the student model
- Experiments demonstrate that our approach is slightly better than other state-of-the-art detectors on the GreenApple and MinneApple and our method has a good generalization ability on the Pascal VOC

The organizational structure of this article is as follows: section 2 is related work that introduces the research status of small object detection, small fruit detection, and feature pyramid network and its variants.  Section 3 elaborates on our method which mainly contains the overview architecture of our method and the details of each component. Experiments are conducted to validate our method in section 4, including evaluation metrics, experimental setting, ablation studies, experiments results, visualization, and qualitative analysis. The discussion and conclusion are presented in section 5.

## 2. Related Work

### 2.1. Small Object Detection

Due to the small objects with low shares in many datasets, the mean average precision of small objects had poor performance. Data enhancement, feature superresolution, and multiscale features are used to capture adequate small object information. To increase the small object's resolution that is similar enough to real large objects, Li et al. [29] proposed a perceptual generative adversarial network by narrowing the representation differences between small objects and large objects to alleviate the above problems. Noh et al. [18] designed a new feature-level superresolution method with a supervisor signal to reduce the loss of the feature generator. However, simulated features are generated with the help of GAN, and the differences between features from the generator and the original feature maps affect the detection performance. Although the GAN-based methods can generate high-resolution images, the artifact features and fabricate fake textures lead to false positives. Moreover, data augmentation is also applied to optimize small object detection performance. Kisantal et al. [30] analyzed the reason for low performance and proposed an augmentation type by copying-pasting small objects multiple times to improve performance. Cui et al. [19] proposed a context-aware module to fuse multiple scale feature maps and the feature receptive field with the help of dilated convolution. Although some achievements have been made in the detection of small targets, the further research about the small object is still needed compared with the precision of medium and large objects.

### 2.2. Small Fruit Detection

Recently, small fruit detection algorithms have achieved remarkable achievements. A series of object detection methods based on deep learning containing single-stage detectors and two-stage detectors are transferred into fruit detection. Hussain et al. [31] proposed a deep convolutional neural network to automatically distinguish similar fruits and vegetables with difficult real-world scenarios. To detect and distinguish the graspable and ungraspable apples, an improved YOLOv5 was devised via BottleneckCSP, SE module, and bonding fusion mode of feature maps by Yan et al. [32]. These above methods are mainly aimed at the target fruits with a large color difference between the target fruits and the background such as orange or red. For the target fruits with similar colors between the fruit skin and the background, the detection precision is slightly lower. Therefore, the precision of small green fruits requires further improvement, which attracts the attention of scholars from home and abroad. Jia et al. [33] proposed an optimized Mask R-CNN to detect green overlapped apples. With the emergence of the attention mechanism and Transformer [34], the self-attention mechanism brings new inspirations to fruit detection. To detect immature/mature apples, the canopy-attention-YOLOv4 [35] was designed by introducing a convolutional block attention module to the generic YOLOv4 detector. A green pepper detection method [36] was proposed based on YOLOv4-tiny, which incorporates an attention mechanism and the idea of multiscale prediction. However, Transformer-based methods require high memory and computing resources [37], which cannot meet the requirements of lightweight in fruit detection.

### 2.3. Feature Pyramid Network and Its Variants

Feature pyramid network was first proposed to incorporate shallow-level features and high-level semantic information. The shallow-level features contain rich texture information, edge information, etc. and the high-level features involve a lot of semantic information. To enhance the information flow, Liu et al. [38] proposed a path aggregation network by bottom-up feature fusion structure to enhance all feature hierarchy with accurate localization signals in lower layers. Tan et al. [39] designed a weighted bidirectional feature pyramid network to fast and ease incorporation of multiplescale feature maps and also devised a compound scaling method for backbone, feature network, and box/class prediction networks. Moreover, a recursive feature pyramid architecture is designed by Qiao et al. [40] to incorporate extra feedback connections from FPNs into the bottom-up backbone layers. These feature fusion structures are mainly fused by extracting feature maps of different layers in the backbone network. However, the feature fusion structure does not take into account the characteristics of its fused features. Specifically, taking small apple detection as an example, the information that can be mapped to small-scale targets gradually decreases or even disappears with the increase of the network layer. This is not conducive to the detection of small-scale apples. Therefore, considering the imbalance of feature information at different sizes, we will alleviate the information imbalance of small apples from two perspectives: balancing information of different scales in the multiresolution feature layers of FPN and constructing high-resolution feature mapping to locate apples.

## 3. Materials and Methods

In this section, the materials are introduced including three dataset benchmarks of GreenApple, MinneApple, and Pascal VOC first. Then, the overview of the balanced feature pyramid network is given. Next, the three components of our method are elaborated as follows.

### 3.1. Materials

Experiments are conducted on the GreenApple, MinneApple [41], and Pascal VOC [42]. Note that the GreenApple dataset is constructed for small green fruit detection and segmentation. MinneApple is a public dataset for small fruit detection and segmentation to validate the effectiveness of our proposed method. The GreenApple dataset is described in detail as follows. Pascal VOC is also a public dataset for general object classification and detection to validate the generalization.

GreenApple is a new green apple dataset, named GreenApple, containing 1361 images and 7137 apple instances. The image acquisition device is a Canon EOS (Electro-Optical System) 80D SLR (Single-Lens Reflex) camera from 8 : 00 to 22 : 00. The collected images are stored in JPEG format and saved with a resolution of 6000 × 4000 pixels. The location of the image collection is the Longwangshan apple production base of the Fushan District, Yantai City, Shandong Province. The latitude and longitude coordinates of the image collection site are 121°3′ east longitude and 37°4′ north latitude. The apple category is Gala. All images from multiple perspectives with varying illumination and weather conditions are collected. These images cover multiple fruit patterns such as single apple without occlusions, branches or leaf occlusion, and overlapped fruits.

**Figure 2:**
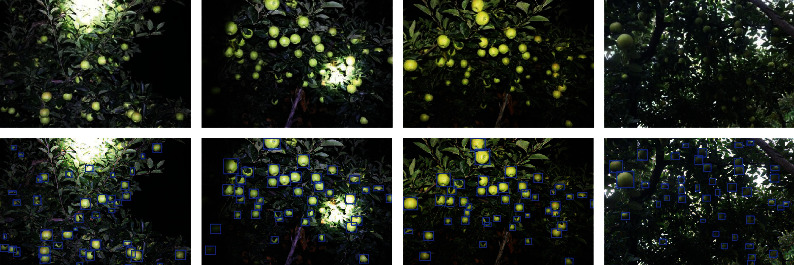
Some original images and notated results on the GreenApple dataset. The top is original images; the bottom is notated results.

The boundaries of the fruit skin and the background are labeled using LabelMe [43]. These images are resized to 600 × 400 pixels and normalized by the mean and variance of all images in our dataset. The original images and notated results are presented in Figure 2. Statistically, according to the pixels occupied by each apple, the percentage of the small, medium, and large green apples of GreenApple dataset are 43.4%, 38.7%, and 17.8%, respectively. Lastly, the GreenApple Dataset is randomly divided into a training set and a test set based on the ratio of 7 : 3. The number and proportion of apple for small, medium, and large scales, as well as the total number of images and instances of apple, are introduced in Table 1.

MinneApple [41] is a public apple dataset for apple detection and segmentation. It contains a training set publicly and a test set privately. The training set contains 670 images and 28183 apple instances to train and test the method. We randomly divide training into a training set and a test set by the proportion of 9 : 1. According to the apple area occupied by pixel number, the division results are presented in Table 2. Note that this dataset does not contain large-scale apples.

Pascal VOC [42, 44] is a public dataset for classification and detection. It consists of Pascal VOC 2007 and Pascal VOC 2012, containing 20 classes. Pascal VOC 2007 and Pascal VOC 2012 trainval sets are considered as the training set, containing 16551 images. The test set of Pascal VOC 2007 is viewed as the test set, including 4952 images to assess our method. The 47223 boxes and 14976 boxes are included in the training set and test set, respectively. Pascal VOC is similar to the GreenApple dataset containing small-, medium-, and large-scale objects. The number of bounding boxes with small, medium, and large scales in Pascal VOC 2007 and 2012 are counted as follows in Table 3.

### 3.2. Overview of BFP Net

Typical FPN incorporates shallow-level feature maps and high-level semantic information in an element-by-element addition manner through the top-down architecture with lateral connections. For multiresolution features from different layers of FPN, the information characteristics between features are ignored. Specifically, shallow features contain the information mapped into all-scale apples, while high-level features mainly are mapped into apples of medium and large scales. In other words, there is an information imbalance in feature mapping for multiple-scale fruits. In addition, due to small physical sizes in the real world and small green apples with the closeness of color between the fruit skin and the background under the orchard environment, these greatly increase the locating and recognition difficulty of small apples.

**Figure 3:**
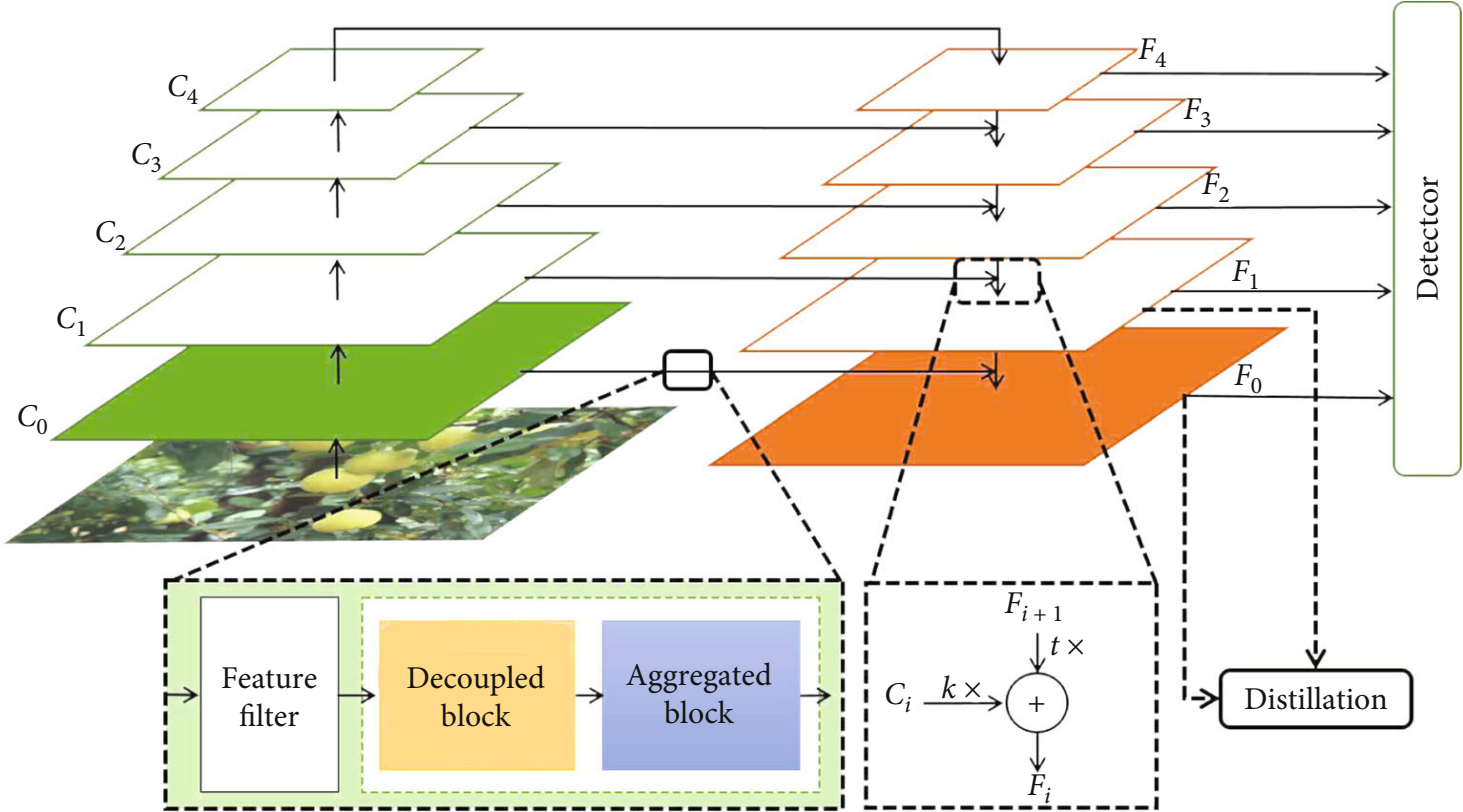
The overview of balanced feature pyramid network (BFP Net). The *C*_*i*_(*i* = 0, 1, 2, 3, 4) refers to the input of FPN from the backbone of ResNet50 and the *F*_*i*_(*i* = 0, 1, 2, 3, 4) denotes the output of FPN. The top 4 layers of FPN are vanilla FPN layers. To balance the multiscale information on different layers, an element-wise addition feature fusion is replaced by a weight-like element-wise addition feature fusion architecture in the vanilla FPN. A new extended layer before pooling is embedded into vanilla FPN to remain a high-resolution feature. Here *C*_0_ and *F*_0_ represent the input and an output of the extended layer. A decoupled-aggregated module with a feature filter is devised to extract content-aware and spatial-aware information. A Kullback-Leibler distillation mechanism is applied to transfer favorable knowledge from *F*_1_ to *F*_0_. Finally, the pyramid-shaped features (*F*_0_, *F*_1_, *F*_2_, *F*_3_, and *F*_4_) are fed to the detector to detect.

To relieve the imbalance problem and difficulty of locating of small apple detection in an unstructured complex orchard environment, a balanced feature pyramid network is proposed with a weight-like feature fusion architecture and a decoupled-aggregated module with a Kullback-Leibler distillation loss for small apple detection, as shown in Figure 3.

Specifically, assuming that the feature maps of the original FPN structure are denoted by *C*_*i*_(*i* = 1, 2, 3, 4) in the bottom-up structure and *F*_*i*_(*i* = 1, 2, 3, 4) in the top-down structure, whose weight parameters are represented by *k* and *t*, respectively. Meanwhile, a new extended feature map *C*_0_ with high resolution is introduced from the output of conv1 in the ResNet50, as shown in Table 4. This extended feature map differs from the extended feature of EFPN, and *C*_0_ contains more spatial and content information due to its high resolution. To balance spatial information and content information and alleviate the problem of difficulty of locating small apple detection, a decoupled-aggregated module is designed on this extended layer. To reduce the difference between an extended feature *F*_0_ and the feature maps *F*_1_, *F*_1_ and *F*_0_ are viewed as a teacher model and a student model, respectively. With the help of the distillation mechanism, a Kullback-Leibler distillation loss is introduced to transfer knowledge. The overview of our proposed method is shown in Figure 3.

### 3.3. Weight-Like Feature Fusion Architecture

The mean average precision of small object detection is about half of that of large objects with state-of-the-art models in recent years [12]. Small green apples also have similar problems. By investigating the characteristics of the different level feature maps, it can be discovered that shallow features have ample information about small apples. However, an element-wise addition feature fusion with the proportion of 1 : 1 at different levels ignores the imbalance problem of multiscale apple information on the different FPN feature layers.

**Figure 4:**
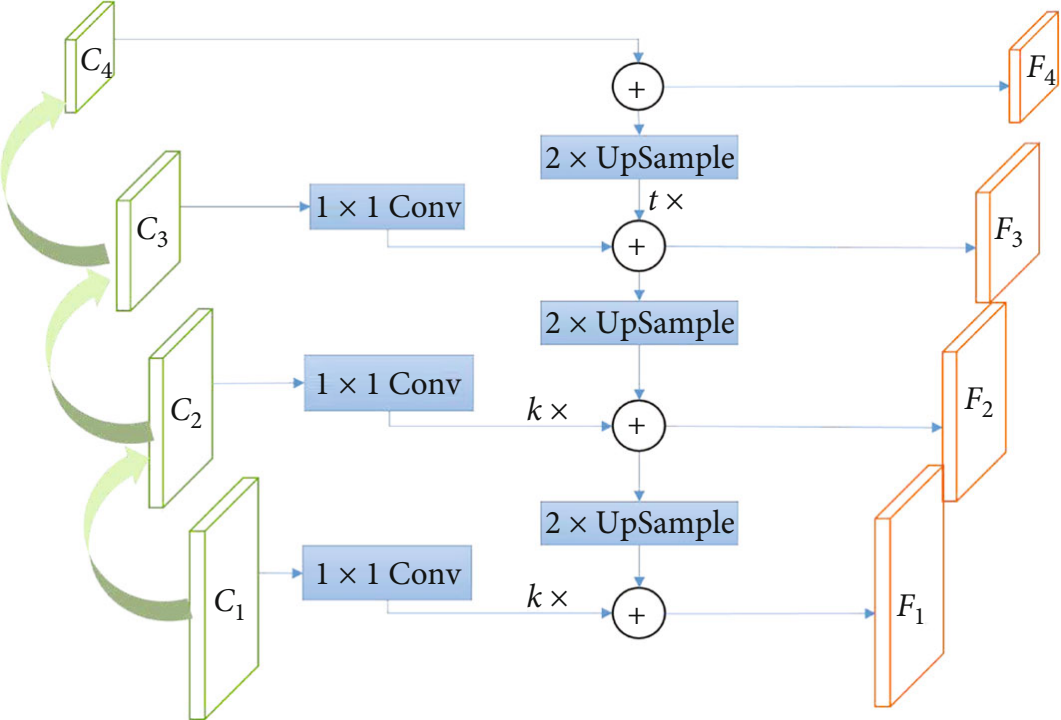
The diagram of weight-like.

Enlightened by the different sensitivities of feature maps on different layers for different scales of apples, a weight-like feature fusion architecture is devised in Figure 4. Two hyperparameters are introduced, weight parameter *k* on the lateral connection and parameter *t* on the top-down pathway, respectively. For low-level feature maps, the weight-based feature maps on the bottom-up architecture can be applied to incorporate more features of all-scale apples, especially small apples. While the high-level feature maps, the weight-like feature maps on the top-down structure can extract more semantic information. As shown in Figure 4, *k*× denotes *k* times feature and *t*× represents *t* times feature.

The four features as input are fed to a detector in the original FPN. Assuming that the two features of the high layer are termed as high layer features on the standard FPN and the two features of the shallow layer are treated as shallow layer features. To preserve more feature information, the standard weight of the feature is set to 1. Mathematically, a feature map *F*_*i*_ can be described as

(1) $$\begin{array}{c} F_{i}=\left\{\begin{array}{cc} k\cdot C_{i}+{↑}_{2\times }F_{i+1} & 0<i<3,\\ C_{i}+t\cdot {↑}_{2\times }F_{i+1} & 3\leq i<4, \end{array}\right. \end{array}$$

where *k* is the weight hyperparameter of the lateral connection in the shallow level, *C*_*i*_ represents the feature map of *i*-th lateral connection, ↑_2×_ denotes double upsampling by nearest-neighbor interpolation, and *t* is a weight parameter of the top-down architecture on the high layer. We take the feature layer *F*_2_ of the standard FPN as an example on the benchmark dataset of GreenApple. To facilitate the experiment, we set *k* to 2. The feature *F*_2_ is obtained by adding double the feature layer *C*_2_ and *F*_3_ after double upsampling by nearest-neighbor interpolation in an element-wise addition manner.

### 3.4. Decoupled-Aggregated Module

The original feature pyramid network pays attention to multiple-resolution feature fusion via lateral connections with a top-down structure. However, different levels of features on the FPN architecture have different sensitivities for small, medium, and large apples. One reason is that only high-level features with low resolution are obtained and shallow information is ignored via a series of convolution and pooling operations. Another reason is that the small apples occupy fewer pixels, and small green apples in the growth stage have the characteristic of similar color between the fruit skin and the background. These greatly increase the locating difficulty of small green apples with area < 32^2^. The FPN-based feature fusion architecture can incorporate shallow-level and high-level semantic information, ignoring spatial location information.

The decoupled-aggregated module with a feature filter is composed of a decoupled block and an aggregated block. The left is a feature filter to refine the feature. The middle is a decoupled block, where the spatial-aware branch of the top is applied to complement spatial location information and the content-aware branch of the bottom is adopted to capture global content information. The right is an aggregated block to incorporate features. Note that PWC refers to point-wise convolution.

To address the information imbalance problem between small apples and large apples, a new extended layer is also introduced to the FPN before the pooling operation, enlightened by the extended feature pyramid network [26]. The source of this extended feature is shown in Table 4. This extended feature layer is obtained after a convolution operation and before pooling in the ResNet50. However, this new extended feature not only contains abundant multiple-scale apple information but includes some noises. These noises would negatively impact the apple recognition and detection. Therefore, a feature filter is first introduced to discard disturbing noises and adjust the number of channels.

**Figure 5:**
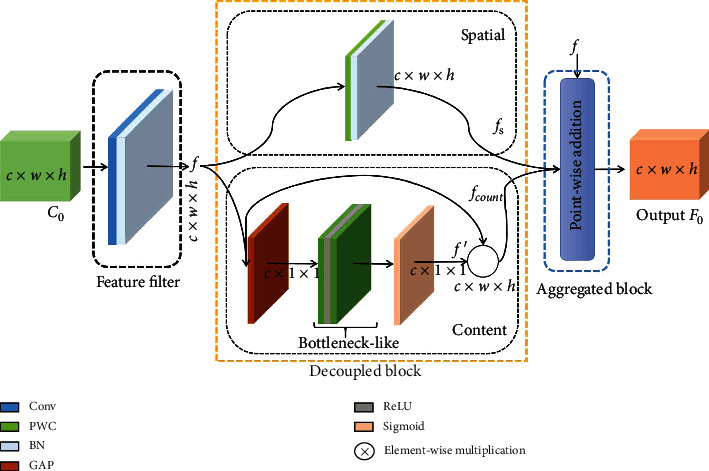
Decoupled-aggregated module.

Inspired by the decoupling principle [45, 46], a decoupled-aggregated module is devised to complement spatial information. The extended feature map can be decoupled into a content-aware feature and a spatial-aware feature. Point-wise addition is applied to aggregate features. This architecture synthesizes spatial information and content information of images. The overview structure of the decoupled-aggregated feature block is shown in Figure 5. The decoupled-aggregated module is elaborated on as follows.

#### 3.4.1. Feature Filter

For the feature filter, a convolution operation with a batch normalization on the extended layer is utilized to reduce noise interference. Specifically, assuming that the input and output of the extended layer are *C*_0_ and *F*_0_, a convolution operation with a kernel size of 3×3, a stride of 2, and a padding of 1 are applied to filter interference noises. Next, a batch normalization is utilized to smooth features and obtain feature *f*.

#### 3.4.2. Decoupled and Aggregated Block

For the decoupled-aggregated block, this module consists of a decoupled block and an aggregated block. The decoupled block can decompose a feature into two features, namely, a spatial-aware feature and a content-aware feature. Then, the aggregated block incorporates features in a point-wise addition manner. The decoupled module and aggregated module are described in detail as follows.

For the spatial-aware branch, a point-wise convolution (PWC) with a normalization layer is adopted to capture a feature map *f*_*s*_ with high resolution that can preserve spatial location information through two convolution operations with the normalization layer. Although the PWC ignores some spatial information [47], a high-resolution feature without pooling operation still preserves abundant spatial information [48].

For the content-aware branch, enlightened by the squeeze and excitation idea in SENet [49] and attention mechanism [34], a global average pooling is first used to transform the feature shape from *c* × *h* × *w* to *c* × 1 × 1, which enlarges the receptive field of the feature map from local to global. In other words, this operation is equivalent to an attention mechanism that can capture abundant global contextual information, which breaks the limitation that convolution only extracts local information. Next, a bottleneck-like architecture is utilized to extract features from the global receptive field. The bottleneck-like structure consists of two point-wise convolutions and a ReLU. Specifically, a point-wise convolution with a dimension reduction factor *γ* is applied to obtain feature information. The dimension reduction factor is applied to squeeze the channel number. A ReLU is an activation function that prevents the gradient from disappearing. A point-wise convolution with channel *c* is utilized to stimulate more feature information, regulate channel number, and enhance feature representation capacity. A sigmoid function is adopted to rescale feature maps. Finally, a new feature map *f*′ can be obtained. The *f*′ is multiplied with the input feature *f* of the content-aware branch to obtain a content-based feature map *f*_cont_.

For the aggregated block, a point-wise addition is applied to aggregate features with spatial-aware, content-aware, and output of a residual structure on the decoupled block. Finally, the output of aggregated block can be obtained to feed to the detector.

Mathematically, the decoupled architecture can be described as follows. The input of the decoupled block is defined as *f*. *f*_*s*_ and *f*_cont_ are viewed as outputs of the spatial-aware branch and content-aware branch. The output *F*_*DA*_ of decoupled-aggregated can be formulated as

(2) $$\begin{array}{c} F_{\text{DA}}=f+f_{s}+f_{\text{cont}.} \end{array}$$

The feature map *F*_0_ can be described as

(3) $$\begin{array}{c} F_{0}=F_{\text{DA}}+F_{1}, \end{array}$$

where *F*_1_ is the feature map from the FPN; *f*_*s*_ is obtained through a point-wise convolution operation with a kernel size of 1×1 and a normalization operation. *f*_cont_ is captured by the squeezing operation Sq(·) based on a global average pooling and exciting operation *Ex*(·) based on the bottleneck-like structure and a sigmoid. Specifically, Sq(·) is represented as follows:

(4) $$\begin{array}{c} z_{c}=\text{Sq}\left(f\right)=\frac{1}{H\times W}\overset{H}{\underset{i=1}{\sum }}\overset{W}{\underset{j=1}{\sum }}f_{c}\left(i,j\right), \end{array}$$

where *H*, *W*, and *C* refer to height, width, and channel number, respectively. *f*_*c*_(*i*, *j*) denotes the feature value of the *c*-th channel, *i*-th row, and *i*-th column. *z*_*c*_ is the global average pooling operation of the *c*-th channel.

*Ex*(·) is formulated as

(5) $$\begin{array}{c} f^{\prime }=\text{Ex}\left(z,w_{1},w_{2}\right)=\sigma \left(w_{2}\delta \left(w_{1},z\right)\right), \end{array}$$

where *σ*(·) denotes a sigmoid function, *δ*(·) refers to activation function of ReLU, *w*_*i*_ refers to the weight of point-wise convolution operation of kernel 1×1 in the content-aware branch, respectively, and where *w*_1_ ∈ *R*^(*γC* × *C*)^ refers to the weight of the first convolution and *w*_2_ ∈ *R*^(*C* × *γC*)^ is the weight of the second convolution. *z* represents the output of Sq(*f*). Finally, the output of the content-aware branch is calculated as follows:

(6) $$\begin{array}{c} f_{\text{cont}}=f⊗f^{\prime }, \end{array}$$

where ⊗ denotes the element-wise multiplication operator.

### 3.5. Kullback-Leibler Distillation Loss

Although the feature map on the extended layer has been filtered and underwent decoupled and aggregated operations, there will still be a few noise information. To make the distribution more consistent between feature map *F*_1_ and *F*_0_, a Kullback-Leibler (KL) divergence loss function is introduced to relieve the loss between features. The KL divergence *D*_KL_ can be described as

(7) $$\begin{array}{c} D_{\text{KL}}\left(p\left\|q\right.\right)=\overset{N}{\underset{i=0}{\sum }}p\left(x_{i}\right)\left(\log\frac{p\left(x_{i}\right)}{q\left(x_{i}\right)}\right), \end{array}$$

where *p* and *q* are probability distribution of *F*_1_ and *F*_0_. The *N* refers to the product of batch size, channel, width, and height of feature *F*_1_ or *F*_0_. The *x*_*i*_ refers to the feature value of *i*-th position. *D*_KL_ calculates the difference between probability *p* and probability *q*.

The smaller the divergence value, the closer the relationship between probability *q* and probability *p* is. That is, the probability distribution of *F*_0_ is closer to that of *F*_1_. *p* and *q* can be described as

(8) $$\begin{array}{c} p=\text{softmax}\left(F_{1}\right),\\ q=\log\left(\text{softmax}\left(F_{0}\right)\right). \end{array}$$

where softmax(·) can be formulated as follows:

(9) $$\begin{array}{c} \text{softmax}\left(x_{i}\right)=\frac{\exp\left(x_{i}\right)}{\sum_{c=1}^{C}\exp\left(x_{c}\right)}, \end{array}$$

where *x*_*i*_ represents the *i*-th feature value of *F*_1_ or *F*_0_; the *c* refers to the *c*-th channel where *x*_*i*_ is located. *C* is the channel number. The exp(·) function is the *x* power of *e*.

However, the KL loss can transfer knowledge between *F*_0_ and *F*_1_ via the back-propagation algorithm. Therefore, to prevent the adverse interference of the KL loss to our method, the distillation mechanism is introduced as a supervisor signal to supervise KL loss. Given that the distillation temperature *T*, the feature map *F*_1_, and an extended feature map *F*_0_ are regarded as the teacher and student models, respectively. Mathematically, softmax_KD_(·) can be optimized as follows:

(10) $$\begin{array}{c} \text{softma}{\text{x}}_{\text{KD}}\left(x_{i},T\right)=\frac{\exp\left(x_{i}/T\right)}{\sum_{c=1}^{C}\exp\left(x_{c}/T\right)}, \end{array}$$

where *x*_*i*_ represents the *i*-th feature value of *F*_1_ or *F*_0_; the *c* refers to the *c*-th channel. *C* is the channel number.

The final KL distillation loss can be formulated as follows:

(11) $$\begin{array}{c} L_{\text{KL}-\text{KD}}=D_{\text{KL}-\text{KD}}\cdot T^{2}, \end{array}$$

where *D*_KL−KD_ represents KL loss based on equation (7) and equation (10).

In this paper, we adopt the Faster R-CNN [27] as the baseline, as an example. The total loss Loss can be described as follows:

(12) $$\begin{array}{c} \text{Loss}=L_{\text{frcnn}}+L_{\text{KL}-\text{KD}},\\ L_{\text{frcnn}}\left(p_{i},t_{i}\right)=\frac{1}{N_{\text{cls}}}\underset{i}{\sum }L_{\text{cls}}\left(p_{i},p_{i}^{*}\right)+\varepsilon \frac{1}{N_{\text{reg}}}\underset{i}{\sum }p_{i}^{*}L_{\text{reg}}\left(t_{i},t_{i}^{*}\right), \end{array}$$

where *L*_cls_ represents the classification loss of cross entropy and *L*_reg_ refers to the regression loss of smooth L1 loss. *p*_*i*_ is the predicted probability of anchor *i*. *N*_cls_ is the minibatch size and *N*_reg_ refers to the number of anchor locations. *ε* represents a balancing weight. If the anchor is positive, the ground-truth label *p*_*i*_^∗^ is 1; otherwise, *p*_*i*_^∗^ is 0. *t*_*i*_ is a vector that represents the coordinate of the predicted bounding box. *t*_*i*_^∗^ is the vector representing the coordinate of the ground-truth bounding box.

## 4. Results

### 4.1. Evaluation Metrics

In this paper, we adopt the precision (*P*) and recall (*R*) [50] as the evaluation indexes. The *P* and *R* can be formulated on a certain intersection over union (IoU) [51] threshold as follows:

(13) $$\begin{array}{c} P=\frac{\text{TP}}{\text{TP}+\text{FP}},\\ R=\frac{\text{TP}}{\text{TP}+\text{FN}}, \end{array}$$

where TP, FP, and FN refer to true positives, false positives, and false negatives, respectively. The mean average precision (AP) and mean average recall (AR) under the IoU = [0.5 : 0.05 : 0.95] are applied to evaluate the methods. To more clearly present the performance of objects at different scales, the average precision of small-scale, medium-scale, and large-scale objects are defined as AP_S_, AP_M_, and AP_L_, respectively. The average recall of small-scale, medium-scale, and large-scale objects are set to AR_S_, AR_M_, and AR_L_, respectively.

For the GreenApple, MinneApple, and Pascal VOC dataset, considering the proportion of small, medium, and large objects and artificial selection of a single IoU can affect the quality of the method assessment. The mean average precision (AP) and recall (AR) at IoU = [0.5 : 0.05 : 0.95] are reported. Moreover, AP_50_ and AP_75_ are also introduced to evaluate the performance of detection. In addition, we use #params to comprehensively measure the parameter quantity.

### 4.2. Implementation Details

Our approach and ablation experiments are implemented with a Faster R-CNN detector, where ResNet50 is applied as the backbone. All models are trained in a single NVIDIA 1080Ti GPU using PyTorch [52]. The original Faster R-CNN is first trained as the baseline. An SGD optimizer is utilized with the weight decay of 5e-4 and a momentum of 0.9. Our method is trained based on 12 epochs. The step learning rate scheduler is applied to a learning rate adjustment strategy of our method, where the step size is set as 3. The aspect ratio size is set as [0.5, 1.0, and 2.0]. For the hyperparameters, the weight values *k* and *t* are both set to 2 to facilitate the experiment. The dimension reduction factor *γ* is set to 0.2. The distillation temperature *T* is set to 103. Further, to validate our method, experiments are conducted compared with state-of-the-art methods on the MinneApple and Pascal VOC.

### 4.3. Ablation Studies

Ablation experiments are conducted on the GreenApple to validate each component and analyze the contributions of each component. Due to the limitation of computer computing resources, all experiments are based on the Faster R-CNN with the backbone of ResNet50 and FPN. Models are trained and tested using the training set and test set of GreenApple. Experimental results are presented in Table 5. It can be observed that the AP_S_ and AR_S_ of baseline on small apples are 45.7% and 55.1% under the IoU = [0.5 : 0.05 : 0.95], respectively. Further, the precision and recall of our method on small apple detection are 47.0% and 57.2%, increased by 1.3% and 2.1%, respectively. When only a weight-like feature fusion architecture is adopted, the AP_S_ and AR_S_ are 46.1% and 55.7% increased by 0.4% and 0.6% for small apple detection, respectively. When a weight-like feature fusion architecture and a decoupled-aggregated module are adopted at the same time, the precision and recall are 46.7% and 56.9%, increasing by 1.0% and 1.8%, respectively. To improve the stability during training, a distillation loss improves the precision by 0.3% for small apples. It can be concluded that each block can improve the precision of small apple detection.

The bigger the weight, the better? The performance of our network is evaluated for different weight parameters with a decoupled-aggregated module and a KL distillation loss, as shown in Table 6. We assume that *k* and *t* are set from 1 to 4 to evaluate the precision and recall of different scales of apple under different parameters. It is noteworthy that *k* and *t* are two different hyperparameters. In this paper, we adopt same values and take integer values to simplify the experiment to verify the validity of our method. It can be observed that AP and AP_S_ do not increase with the magnified weight. When *k* and *t* take 2, AP_S_ and AR_S_ get the optimal value of 47.0% and 57.2%, increased by 1.3% and 2.1% compared with the baseline of Faster R-CNN. It can be seen that our method can improve small apple detection performance in Table 6. Note that when the weight is set as 1, our method is equivalent to the weight-like feature fusion architecture. While the precision for small apple detection is improved, the precision of medium and large apple detection is slightly lower with the weight of 2. Although AP_M_ and AP_L_ are slightly smaller. Finally, the weight is empirically assigned as 2 for optimal performance considering the mean average precision of the small, medium, and large scales comprehensively. Therefore, the weight-like feature fusion structure can effectively improve the precision of small apple detection.

Importance of decoupled block: the performance of the spatial branch and content branch are tried to assess, respectively. Experiments are conducted for every branch with the weight-like feature fusion structure, feature filter, and aggregated feature module. Experimental results are presented in Table 7. Only spatial-aware branches or content-aware branches can improve the performance of our proposed model by 1.1% and 0.6%. The precision can be improved by 1.3% using two branches of the spatial-aware and content-aware branches. It can be observed that the decoupled-aggregated feature block with the single spatial feature or single content feature can improve slightly our model. Compared with the content-aware branch, the spatial-aware branch can significantly improve the precision of small apple detection. Firstly, it benefits from the fact that the input of the extended layer feature is a high-resolution feature; secondly, the output of the spatial sensing branch only goes through the two convolution operations with the normalization layer and does not go through the downsampling of pooling, which can retain more feature information that can be mapped to small apples. Thus, it can be concluded that spatial architecture can play a greater role than the content-aware branch. The spatial branch is necessary for capturing object structure information via the point-wise convolution without downsampling. Content-aware branch obtains global contextual feature information by global average pooling, which breaks the limitation that convolution only extracts features locally. In contrast, the feature decoupled block and aggregated block can effectively improve the recognition precision of small apple detection. A feature map from the spatial-aware architecture is indispensable for supplying spatial information about the target.

### 4.4. Results on the GreenApple

Some state-of-the-art methods are compared with our method on the GreenApple and the results are shown in Table 8. All comparison models are trained and tested based on MMDetection [53] on a single NVIDIA 1080Ti GPU. The ResNet50 and FPN architecture are utilized as the backbone and neck. Precision and recall are applied as the evaluation indicators. For comparison methods, an SGD optimizer is adopted with an initial learning rate of 0.0025, a momentum of 0.9, and a weight decay of 5e-4. 1× schedule and 12 epochs with 2 images per GPU are applied to reduce required computing resources. The step size and gamma are set to 3 and 0.33, respectively. To analyze the performance of all methods for small, medium, and large apples, the precision and recall are evaluated at different scales based on the evaluation index of IoU = [0.5 : 0.05 : 0.95], IoU = 0.5, and IoU = 0.75. The dimension reduction factor *γ* is set to 0.2. The distillation temperature is set to 103°C.

**Figure 6:**
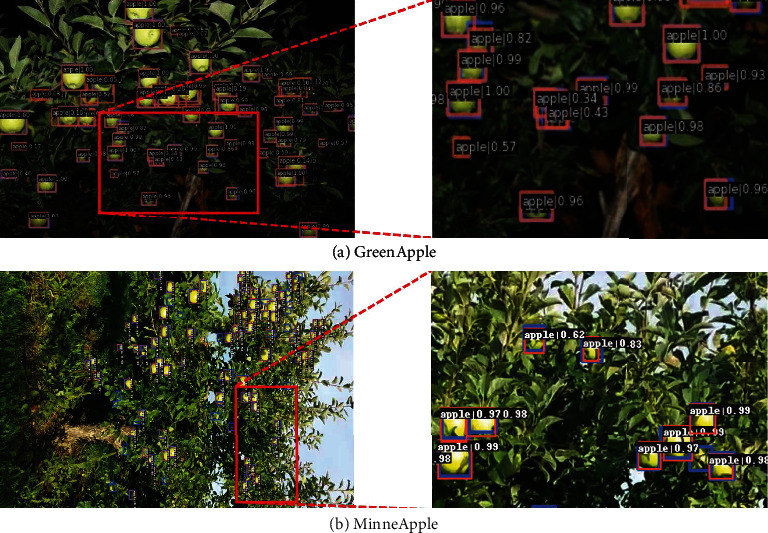
The visualization results of BFP Net on the GreenApple and MinneApple. The left images are prediction results and the right images show the detail of the red rectangular boxes. Note that we rotate the prediction results of MinneApple 90 clockwise. (a) GreenApple, (b) MinneApple.

In Table 8, experimental results are presented on the GreenApple. Compared with Faster R-CNN based on the ResNet50, AP_S_ and AR_S_ of our method can achieve 47.0% and 57.2% increased by 1.3% and 2.1%, respectivey. It can be observed that BFP Net outperforms the Faster R-CNN and its variants for small apple detection. To better observe the detection performance of BFP Net, we present the prediction results of an image, as shown in Figure 6(a). The figure on the left shows the overall prediction results of the image and the figure on the right shows the local results corresponding to the red rectangle. Therefore, experimental results illuminate that BFP Net can improve the precision of small apple detection. The AP_S_ and AP on the GreenApple test set can reach 47.0% and 62.3%, respectively.

In addition, CARAFE, SABL, Dynamic R-CNN, PISA, Mask R-CNN, and Groie are applied to compare the effectiveness of BFP Net for small apple detection. The AP_S_ of our method is increased by 3.2%, 0.3%, 1.4%, 2.7%, 0.2%, and 1.7% compared with CARAFE [54], SABL [55], Dynamic R-CNN [56], PISA [57], Mask R-CNN [58], and Groie [59]. Meanwhile, the AR_S_ can be better than other state-of-the-art methods based on small apples, i.e., 57.2% vs 53.0% and 57.2% vs 54.8%, respectively. Therefore, our method is better than other state-of-the-art models for small apple detection.

**Figure 7:**
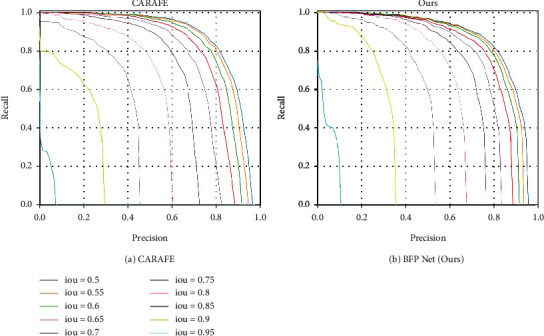
P-R curve of our model and CARAFE based on all thresholds of [0.5 and 0.95] on the GreenApple. (a) CARAFE, (b) ours.

In Figure 7, the precision-recall (P-R) curve of CARAFE and BFP Net are shown on the GreenApple test set. The area enclosed by the P-R curve and the two coordinate axes can be applied to evaluate the quality of a model. When the P-R curve of one model is surrounded by the precision-recall curve of another model, the latter model is better than the former model [50]. The P-R curves are drawn based on IoU = [0.5 : 0.05 : 0.95] about CARAFE and our method firstly. It can be found that the area enclosed by P-R curves and two coordinate axes under the certain IoU threshold of our method is larger than that of CARAFE. It can be concluded that with the increase of IoU, the effect of the model is more significant. Compared with CARAFE, our performance can be better than other methods when the IoU threshold is greater than 0.6.

**Figure 8:**
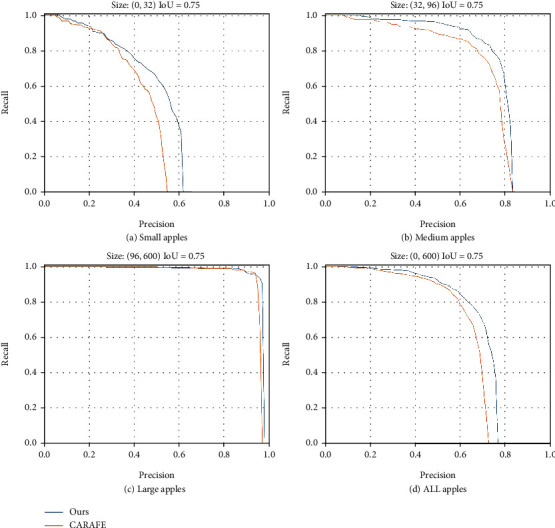
Comparison precision and recall of BFP Net and CARAFE with IoU = 0.75 on the GreenApple test set. (a) Small apples, (b) medium apples, (c) large apples, and (d) all apples.

To more clearly show the effectiveness of our proposed model at a specific threshold, 0.75 is adopted for the IoU threshold as an example to draw the P-R curves of BFP Net and CARAFE for different scales of fruits, as shown in Figure 8. Blue and orange lines are the effects of BFP Net and CARAFE. From this picture, BFP Net has a significant effect on apple detection, especially on small and medium apple detection.

To evaluate the model more comprehensively, we also use the #params to evaluate the performance of our method. The evaluation results of parameters are shown in Table 9. In Table 9, it can be observed that the #params of BFP Net is 42.55 M. Compared with the baseline of Faster R-CNN, the #params of our method increased by 1.43 M. Therefore, we can conclude that the BFP Net can improve the detection performance of small apple detection without increasing a large number of parameters.

### 4.5. Results on the MinneApple

To validate our method, MinneApple is applied to assess BFP Net and other state-of-the-art methods. The results are presented in Table 10. It can be found that our method can outperform other state-of-the-art methods. The AP_S_ and AR_S_ of our method are 42.2% and 48.2%, increased by 1.0% and 1.3%, respectively, compared with the baseline of Faster R-CNN. Note that AP_L_ and AR_L_ are -1 because there is no large-scale apples. A prediction image is presented in Figure 6(b). The orange and blue bounding boxes represent the prediction and ground-truth bounding boxes, respectively. By comprehensively analyzing the prediction results and visualization results, we can concluded that our method is applicable to small apple detection in a real orchard environment.

We also compared BFP Net with many state-of-the-art models, and the results are presented in Table 10. It can be observed that the AP_S_ is increased by 1.3%, 1.7%, 3.6%, 0.3%, 1.3%, and 1.2%, compared with CARAFE [54], SABL [55], Dynamic R-CNN [56], PISA [57], Mask R-CNN [58], and Groie [59]. Meanwhile, AP_M_, AP_L_, and AP of BFP Net outperform some state-of-the-art methods in Table 10. Similarly, AR_S_, AR_M_, AR_L_, and AR are better than the comparison models. Overall, BFP Net can outperform some state-of-the-art methods on the MinneApple.

### 4.6. Results on the Pascal VOC

We also verified the generalization ability by the generic object detection dataset of Pascal VOC. The experimental results are presented in Table 11. In Table 11, experimental results of our proposed method and baseline show that the AP_S_ and AR_S_ of our method can achieve 35.6% and 48.9%, increased by 8.5% and 10.4%, respectively, based on IoU = [0.5 : 0.05 : 0.95], compared with Faster R-CNN. The AP_M_ and AR_M_ can reach 42.9% and 57.1%, respectively. As a whole, BFP Net outperforms the baseline of Faster R-CNN on the Pascal VOC. Meanwhile, we also validated and compared our method with some state-of-the-art models in Table 11. It can be observed that the *A*P_S_, AP_M_, AR_S_, and AR_M_ of BFP Net are better than some state-of-the-art methods, which illustrates that our method can be better than some state-of-the-art models on the small-scale objects and medium-scale objects. However, the AR_L_ and AR_L_ are 54.3% and 82.1%, respectively, which is better than that of CARAFE [54], Dynamic R-CNN [55], PISA [56], and baseline, but is slightly lower than SABL [57], Mask R-CNN [58], and Groie [59].

For this phenomenon, compared with the apple fruits with a class of apple and shape of spherical in the real orchard environment, we found that the number of categories in Pascal VOC is larger than that of MinneApple/GreenApple and the shapes of samples in each category are complex and diverse, which greatly increase the difficulty of target detection. Although BFP Net is not completely superior to the comparison models, as a whole, the BFP Net has a strong generalization ability for detecting the general object, especially for small target detection.

### 4.7. Visualization

To present clear detection results, BFP Net and some state-of-the-art models are visualized based on the GreenApple. The weight file obtained from the training set is applied to validate the model on the GreenApple test set. The visualization results are presented on the image through the orange rectangular box annotated with category and precision. Note that the category and precision of the target are located at the top left of the target rectangle. In addition, the ground-truth bounding box are presented with blue color.

**Figure 9:**
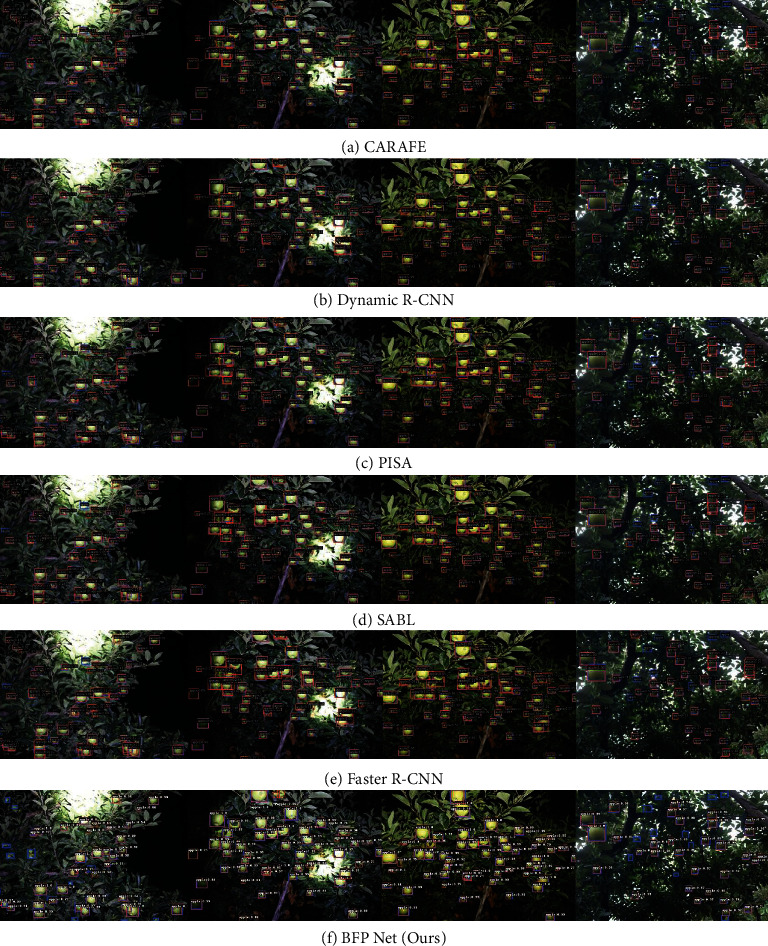
Visualization results of BFP Net and some state-of-the-art methods on the GreenApple. The four image cases in each line represent the four visualization results of corresponding method. The visualization methods contain CARAFE, Dynamic R-CNN, PISA, SABL, Faster R-CNN, and BFP Net (ours) from top to bottom. Note that the blue bounding box refers to the ground-truth bounding box and the orange bounding box represents the prediction bounding box on the GreenApple test set.

Some visualization results about state-of-the-art models, baseline (Faster R-CNN) and our method are shown on the GreenApple, as shown in Figure 9. Specifically, visualization results of CARAFE, Dynamic R-CNN, PISA, SABL, Faster R-CNN (baseline), and BFP Net are presented using 4 cases. From this figure, it can be concluded that many methods can detect small apples with closeness of color between the fruit skin and the background in the real complex orchard environment. Compared with other state-of-the-art methods, our method can effectively improve the precision of small apple detection. It can be concluded that our method is suitable for for small apple detection to handle real-world complexities with varying light conditions, as well as occlusion or overlapped fruits.

### 4.8. Qualitative Results

**Figure 10:**
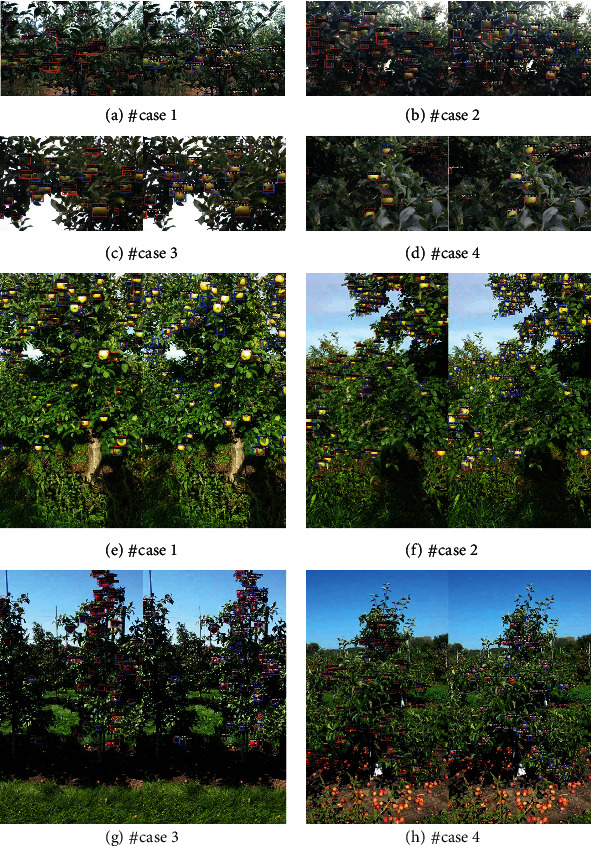
Qualitative results about the CARAFE and BFP Net (ours) on the GreenApple and MinneApple. Each dataset shows four samples for qualitative analysis. Note that the blue bounding boxes are the ground-truth bounding boxes; the orange bounding boxes are the prediction bounding boxes. The (a)–(d) are the results of GreenApple and (e)–(h) are results of MinneApple.

To further validate our method, we qualitatively analyze our method and CARAFE on the benchmark dataset of GreenApple, MinneApple, and Pascal VOC. Four prediction results from GreenApple and MinneApple are selected to analyze the effectiveness of the BFP Net and CARAFE, as shown in Figure 10.

Detection results of GreenApple and MinneApple are shown in Figure 10. It can be observed that precision of the BFP Net is higher than that of CARAFE. Although the apples is small and the color of the fruit skin is similar to the background, small apples can still be recognized in GreenApple. Compared with CARAFE based on ResNet50 with the FPN, BFP Net is better on the GreenApple and MinneApple, especially in small and dense fruit scenes. Overall, our method has strong competitiveness on large public datasets.

In addition, we also analyze the prediction results on the Pascal VOC. The results illustrated that our method has good generalization ability. To make the visualization results of qualitative analysis more consistent with this journal, we only show the visualization results based on the GreenApple and MinneApple in Figure 10.

## 5. Discussion and Conclusion

### 5.1. Discussion

The effectiveness of variants of general object detection and small object detection for small apple detection has been widely used in a series of studies [31–36]. In this article, a balanced feature pyramid network achieved the precision improvement of small apple detection. The balanced feature pyramid network could be directly embedded into existing object detectors to detect small apples, which had great potential for an unstructured orchard environment.

In previous studies, fruit detection methods based on conventional machine learning and deep learning had been widely used. The results of the deep learning method always outperformed machine learning methods [60]. Manually extracted features from a series of small apple detection methods based on machine learning could not be comprehensively acquired [61], which leads to low efficiency and high labor cost. On the other hand, some generic object detection models based on deep learning are not completely suitable for fruit detection, due to small objects with fewer pixels under the complex natural orchard environment and the closeness of color between the fruit skin and the background for small fruits. The precision/accuracy of small objects is half of the large objects [12]. The unbalanced traits between multiple-scale fruits limit the improvement of small target performance. The potential of multiscale information imbalance could not be fully revealed.

Balanced multiple-resolution feature fusion and spatial information presented promising results for small apple detection. Incorporating multiple fine-grained scale apple features and multiple-resolution pyramid features can acquire a better precision for small apple detection. Some studies have studied multiple scale objects [19, 62]. Overall, these methods showed that aggregating multiple scale object features and pyramid features can balance different scale features. The good performances illustrated that balanced different scale features and different characteristic features on the extended layer have the great potential to aggregate more information for small apple detection.

Extended feature extracted abundant traits of multiple-scale apples. The fusion of extended features and pyramid-shaped multiple-scale features aggregated different scale features. The results proved that using the extended feature with high resolution had the potential to improve the precision of small apple identification and detection. In many studies, the authors also extended features on the feature fusion architecture to aggregate multiple-scale features and enhance the strong representation ability of apple features [26].

Moreover, the variations of performance gain between the weight-like feature fusion structure and decoupled-aggregated block with a KL distillation loss can be roughly evaluated. Compared with a limited feature from the backbone on the weight-like feature fusion architecture, an extended feature through decoupled-aggregated block with a KL distillation loss can generate more feature gain to improve the performance of small apple detection. Compared with a lightweight content-aware upsample operator in CARAFE [54], the weight-like feature fusion structure by balancing reliable features from the backbone could acquire more fine-grained fruit traits. Similarly, some artifact features and fabricated fake textures constructed by small object detection methods based on the GAN lead to false positives [18]. It can be concluded that the reliable feature from original images could generate more gains compared with artifact features. For the decoupled-aggregated block with a loss function, an abundant feature from before pooling remains more features of small-scale apple, which can obtain more gain compared with a limited feature. In ablation studies, these views had proved that an unlimited feature was more helpful to optimize small fruit detection performance. The results presented the potential of the balanced reliable feature with abundant information, and more efforts should be made to balance reliable features. In addition, proposal region or sample assign strategy etc. [55–57, 59] for detector were also designed to optimize the precision in many studies. Given the poor performance of small targets compared to large-scale targets, the optimization of target detectors has limitations for small-scale target performance improvement, and the results have been validated.

With the emergence of feature extraction and multiple scale feature fusion techniques, phenotyping features with strong representation ability can be obtained from different aspects. How to balance information features of different scales to recognize and detect multiple-scale apples during the whole process of fruit growth to maturity is of importance. Information balance of multiple aspects and scales provided an effective alternative to balance multiple scale features and spatial/semantic information on the extended layer. The performance of information balance for small apple detection proved that the information balance of different traits was promising. Therefore, different views could be analyzed to assess the effectiveness of small apple detection. The information balance of multiple perspectives will provide more fine-grained information and alleviate the problem of blurred boundaries on small apples, and how to effectively balance the phenotyping traits from multiple views remain a challenging issue. This article provided an efficient choice for information balance of different scale phenotyping features for small apple detection in real complex orchard environments, which could aggregate information from different perspectives to apply to the problem of the closeness of color between the fruit skin and the background of branches and leaves. Last but not least, we directly chose an existing detector to detect small apples but this detector cannot completely apply to the detection of different fruit scales in the whole process of fruit growth. How to optimize the fruit detector and making it suitable for small fruit/vegetable detection is also a potential alternative, which could provide some technical support for yield prediction, picking of mature fruits, and automatic monitoring of agriculture.

### 5.2. Conclusion

In conclusion, balanced apple information of different scales from two perspectives of multiple-scale fruits on the different layers of FPN and a characteristic of an extended feature has a certain effect on reducing the gap of apple information of different scales. The BFP Net presents strong potential to improve the performance of small apple detection. The experimental results also illustrated that a weight-like feature fusion structure can refine multiple-scale feature information. A decoupled-aggregated module can balance spatial-aware information and content-aware information to complement the spatial information of small apples based on an extended layer. The Kullback-Leibler distillation loss make the feature distribution consistent between a high-level feature and extended feature map. The results demonstrate that each component of the balanced feature pyramid network facilitates the improvement of small apple detection precision, in particular, the precision of small apple localization. In addition, this method has the ability to improve the precision without adding a large number of parameters. This method can not only be applied to other small apple detection based on feature pyramid feature fusion architecture but also provide a reference for other fruit/vegetable detection.

## Figures and Tables

**Table 1:** The division results of GreenApple dataset. Note that small apples with area < 32^2^, medium apples with 32^2^ < area < 96^2^, and large apples with area > 96^2^ are counted.

Area	Small	32^2^	Medium	96^2^	Large	Total apples	Total images
Training	2079/42.0%	1	1973/39.9%	0	890/18.0%	4943	953
Test	1022/46.5%	1	787/35.9%	0	384/17.5%	2194	408
Total	3101/43.4%	2	2760/38.7%	0	1274/17.8%	7137	1361

**Table 2:** The division results of MinneApple dataset. Note that small apples with area < 32^2^, medium apples with 32^2^ < area < 96^2^, and large apples with area > 96^2^ are counted.

Area	Small	32^2^	Medium	96^2^	Large	Total apples	Total images
Training	22306/88.3%	0	2943/11.7%	0	0	25249	603
Test	2593/88.4%	0	341/11.6%	0	0	2934	67
Total	24899/88.3%	0	3284/11.7%	0	0	28183	670

**Table 3:** The division results of Pascal VOC. Note that small objects with area < 32^2^, medium objects with 32^2^ < area < 96^2^, and large objects with area > 96^2^ are counted.

Area	Small	32^2^	Medium	96^2^	Large	Total boxes	Total images
Training	5300/11.2%	5	14525/30.7%	4	27389/57.9%	47223	16551
Test	1661/11.1%	3	5077/33.9%	2	8233/54.9%	14976	4952
Total	6961/11.1%	8	19602/31.5%	6	35622/57.2%	62199	21503

**Table 4:** The layer structure of Faster R-CNN, EFPN, and BFP Net (ours). ResNet50 is applied as the backbone of these methods.

Method	Faster R-CNN [27]	EFPN [26]		BFP Net (ours)
Input size	(600, 400)	(600, 400)	(600, 400)	(600, 400)
Stage 1	7 × 7, 64, *s* = 2, pad = 3	7 × 7, 64, *s* = 2, pad = 3		7 × 7, 64, *s* = 2, pad = 3
Output	—	—	—	*C*_0_: (300, 200)
Stage 2	3 × 3 max pool, *s* = 2	—	3 × 3 max pool, *s* = 2	3 × 3 max pool, *s* = 2
	Residual block (×3)	Residual block (×3)		Residual block (×3)
Output	*C*_2_: (150, 100)	*C*_2_′: (300, 200)	*C*_2_: (150, 100)	*C*_1_: (150, 100)

**Table 5:** Performance of each component on the GreenApple test set. Note that D-A refers to the decoupled-aggregated module.

Weight-like	D-A	Loss	AP_S_	AP_M_	AP_L_	AR_S_	AR_M_	AR_L_	AP_50_	AP_75_	AP
			45.7	68.3	88.4	55.1	74.4	92.1	85.9	69.3	62.3
✓			46.1	68.5	87.9	55.7	74.4	91.4	85.8	70.2	62.2
✓	✓		46.7	68.6	86.5	56.9	75.1	90.8	86.8	69.5	62.2
✓	✓	✓	47.0	68.1	87.5	57.2	75.4	91.2	86.7	69.1	62.3

**Table 6:** Performance of different weights on the GreenApple test set. To simplify the experiment, we take the same value for *k* and *t*. Meanwhile, values of *k* and *t* are integers.

*k*	*t*	AP_S_	AP_M_	AP_L_	AR_S_	AR_M_	AR_L_	AP_50_	AP_75_	AP
1	1	46.8	68.4	86.3	56.3	75.0	90.5	86.1	68.7	61.9
2	2	47.0	68.1	87.5	57.2	75.4	91.2	86.7	69.1	62.3
3	3	46.2	68.9	86.8	56.8	75.6	91.0	87.2	70.3	62.7
4	4	46.2	67.1	85.5	56.5	73.8	89.5	86.4	68.1	61.2

**Table 7:** The performance of our method based on different branches.

Spatial	Content	AP_S_	AP_M_	AP_L_	AR_S_	AR_M_	AR_L_	AP_50_	AP_75_	AP
✓		46.8	68.6	87.2	57.6	75.5	91.1	86.9	70.0	62.4
	✓	46.3	68.8	86.2	56.5	75.5	90.5	86.8	69.5	62.2
✓	✓	47.0	68.1	87.5	57.2	75.4	91.2	86.7	69.1	62.3

**Table 8:** Our proposed method and comparison results on the GreenApple test set.

Method	AP_S_	AP_M_	AP_L_	AR_S_	AR_M_	AR_L_	AP_50_	AP_75_	AP	AR
CARAFE [54]	43.8	67.5	86.2	53.0	72.6	89.4	85.3	66.8	60.1	66.4
SABL [55]	46.7	69.6	87.7	55.0	75.3	90.8	86.6	68.3	62.4	68.6
Dynamic R-CNN [56]	45.6	68.5	86.5	53.2	73.6	89.7	85.5	67.8	61.3	67.0
PISA [57]	44.3	68.5	85.1	54.8	84.0	88.3	85.6	67.6	60.9	67.6
Mask R-CNN [58]	46.8	68.6	86.0	54.5	74.1	89.1	86.8	68.7	61.8	67.6
Groie [59]	45.3	68.3	86.7	52.8	73.6	89.6	86.7	68.0	61.3	66.7
Faster R-CNN [27]	45.7	68.3	88.4	55.1	74.4	92.1	85.9	69.3	62.3	68.5
BFP Net	47.0	68.1	87.5	57.2	75.4	91.2	86.7	69.1	62.3	69.7

**Table 9:** Analysis of model parameters.

Method	Input size	#params
CARAFE [54]	(3, 600, 400)	46.73 M
SABL [55]	(3, 600, 400)	41.91 M
Dynamic R-CNN [56]	(3, 600, 400)	41.12 M
PISA [57]	(3, 600, 400)	41.12 M
Mask R-CNN [58]	(3, 600, 400)	43.75 M
Groie [59]	(3, 600, 400)	43.02 M
Faster R-CNN [27]	(3, 600, 400)	41.12 M
BFP Net	(3, 600, 400)	42.55 M

**Table 10:** Comparison results on the MinneApple.

Method	AP_S_	AP_M_	AP_L_	AR_S_	AR_M_	AR_L_	AP_50_	AP_75_	AP	AR
CARAFE [54]	40.9	55.4	-1	46.5	63.9	-1	82.6	37.5	42.0	48.5
SABL [55]	40.5	57.4	-1	46.4	66.3	-1	81.5	37.7	42.0	48.7
Dynamic R-CNN [56]	38.6	53.4	-1	44.7	62.6	-1	81.7	33.9	39.9	46.8
PISA [57]	41.9	57.5	-1	47.9	65.6	-1	84.5	39.3	43.4	49.9
Mask R-CNN [58]	40.9	55.1	-1	46.6	63.8	-1	82.9	37.7	42.0	48.6
Groie [59]	41.0	55.3	-1	46.7	63.9	-1	82.6	38.0	42.3	48.7
Faster R-CNN [27]	41.2	56.3	-1	46.9	65.0	-1	83.1	38.8	42.6	49.0
BFP Net	42.2	58.3	-1	48.2	66.8	-1	84.6	40.6	43.5	50.4

**Table 11:** Our proposed method and comparison results on the Pascal VOC 2007 test set based on the IoU threshold of [0.5 : 0.05 : 0.95].

Method	AP_S_	AP_M_	AP_L_	AR_S_	AR_M_	AR_L_	AP_50_	AP_75_	AP	AR
CARAFE [54]	26.7	40.2	54.0	38.1	49.2	63.5	81.0	54.4	50.2	59.9
SABL [55]	28.5	42.7	57.0	39.8	52.4	67.6	79.3	56.9	52.9	63.7
Dynamic R-CNN [56]	29.8	41.7	53.8	36.2	50.2	63.2	79.8	54.9	50.7	60.0
PISA [57]	27.5	40.4	52.6	38.1	48.8	62.1	80.0	53.3	49.3	58.9
Mask R-CNN [58]	32.0	41.3	57.0	43.3	53.2	67.0	83.6	57.8	53.0	63.7
Groie [59]	29.6	40.7	56.7	39.9	50.2	66.7	83.7	57.3	52.6	62.5
Faster R-CNN [27]	27.1	40.0	51.6	38.5	48.8	62.0	80.1	51.2	48.5	58.9
BFP Net	35.6	42.9	54.3	48.9	57.1	65.9	82.1	56.2	51.4	63.8

## Data Availability

The data used in this study are freely available. Anyone who wants to use the data can contact the corresponding author Weikuan Jia. The author is with the School of Information Science and Engineering, Shandong Normal University, Jinan 250358, China (jwk_1982@163.com).

## References

[1] Zhang W., Chen K., Wang J., Shi Y., Guo W. (2021). Easy domain adaptation method for filling the species gap in deep learning-based fruit detection. *Horticulture Research*.

[2] Ukwuoma C. C., Zhiguang Q., Bin Heyat M. B., Ali L., Almaspoor Z., Monday H. N. (2022). Recent advancements in fruit detection and classification using deep learning techniques. *Mathematical Problems in Engineering*.

[3] Mamdouh N., Wael M., Khattab A. (2022). Artificial intelligence-based detection and counting of olive fruit flies: a comprehensive survey. *Deep Learning for Sustainable Agriculture, Chapter 14*.

[4] Maheswari P., Raja P., Apolo-Apolo O. E., P’erez-Ruiz M. (2021). Intelligent fruit yield estimation for orchards using deep learning based semantic segmentation techniques—a review. *Frontiers in Plant Science*.

[5] Singh P., Kaur A. (2022). A systematic review of artificial intelligence in agriculture. *Deep Learning for Sustainable Agriculture, Chapter 2*.

[6] Koirala A., Walsh K. B., Wang Z., McCarthy C. (2019). Deep learning – method overview and review of use for fruit detection and yield estimation. *Computers and Electronics in Agriculture*.

[7] Ni X., Li C., Jiang H., Takeda F. (2020). Deep learning image segmentation and extraction of blueberry fruit traits associated with harvestability and yield. *Horticulture Research*.

[8] Faiçal B. S., Freitas H., Gomes P. H. (2017). An adaptive approach for uav-based pesticide spraying in dynamic environments. *Computers and Electronics in Agriculture*.

[9] Tang Y., Chen M., Wang C. (2020). Recognition and localization methods for vision-based fruit picking robots: a review. *Frontiers in Plant Science*.

[10] Jia W., Zhang Y., Lian J., Zheng Y., Zhao D., Li C. (2020). Apple harvesting robot under information technology: a review. *International Journal of Advanced Robotic Systems*.

[11] Behera S. K., Sethy P. K., Sahoo S. K., Panigrahi S., Rajpoot S. C. (2021). On-tree fruit monitoring system using iot and image analysis. *Concurrent Engineering*.

[12] Tong K., Wu Y., Zhou F. (2020). Recent advances in small object detection based on deep learning: a review. *Image and Vision Computing*.

[13] Lin T.-Y., Maire M., Belongie S. (2014). Microsoft coco: common objects in context. *European conference on computer vision*.

[14] Liakos K. G., Busato P., Moshou D., Pearson S., Bochtis D. (2018). Machine learning in agriculture: a review. *Sensors*.

[15] Behera S. K., Rath A. K., Mahapatra A., Sethy P. K. (2020). Identification, classification & grading of fruits using machine learning & computer intelligence: a review. *Journal of Ambient Intelligence and Humanized Computing*.

[16] Chaivivatrakul S., Dailey M. N. (2014). Texture-based fruit detection. *Precision Agriculture*.

[17] Gené-Mola J., Gregorio E., Cheein F. A. (2020). Fruit detection, yield prediction and canopy geometric characterization using lidar with forced air flow. *Computers and Electronics in Agriculture*.

[18] Noh J., Bae W., Lee W., Seo J., Kim G. Better to follow, follow to be better: towards precise supervision of feature super-resolution for small object detection.

[19] Cui L., Lv P., Jiang X. (2020). Context-aware block net for small object detection. *IEEE Transactions on Cybernetics*.

[20] Zheng H., Chen J., Chen L., Li Y., Yan Z. (2020). Feature enhancement for multi-scale object detection. *Neural Processing Letters*.

[21] Bai Y., Zhang Y., Ding M., Ghanem B. Sod-mtgan: small object detection via multi task generative adversarial network.

[22] Liang Z., Shao J., Zhang D., Gao L. Small object detection using deep feature pyramid networks.

[23] Lin T. Y., Dollár P., Girshick R., He K., Hariharan B., Belongie S. Feature pyramid networks for object detection.

[24] Wang X., Yu K., Wu S. Esrgan: enhanced super-resolution generative adversarial networks.

[25] Rabbi J., Ray N., Schubert M., Chowdhury S., Chao D. (2020). Small-Object detection in remote sensing images with end-to-end edge-enhanced Gan and object detector network. *Remote Sensing*.

[26] Deng C., Wang M., Liu L., Liu Y., Jiang Y. (2022). Extended feature pyramid network for small object detection. *IEEE Transactions on Multimedia*.

[27] Ren S., He K., Girshick R., Sun J. (2015). Faster r-cnn: towards real-time object detection with region proposal networks. *Advances in Neural Information Processing Systems*.

[28] He K., Zhang X., Ren S., Sun J. Deep residual learning for image recognition.

[29] Li J., Liang X., Wei Y., Xu T., Feng J., Yan S. Perceptual generative adversarial networks for small object detection.

[30] Kisantal M., Wojna J. M., Naruniec J., Cho K. (2019). Augmentation for small object detection. http://arxiv.org/abs/1902.07296.

[31] Hussain D., Hussain I., Ismail M., Alabrah A., Ullah S. S., Alaghbari H. M. (2022). A simple and efficient deep learning-based framework for automatic fruit recognition. *Computational Intelligence and Neuroscience*.

[32] Yan B., Fan P., Lei X., Liu Z., Yang F. (2021). A real-time apple targets detection method for picking robot based on improved yolov5. *Remote Sensing*.

[33] Jia W., Tian Y., Luo R., Zhang Z., Lian J., Zheng Y. (2020). Detection and segmentation of overlapped fruits based on optimized mask r-cnn application in apple harvesting robot. *Computers and Electronics in Agriculture*.

[34] Vaswani A., Shazeer N., Parmar N. (2017). Attention is all you need. *Advances in Neural Information Processing Systems*.

[35] Lu S., Chen W., Zhang X., Karkee M. (2022). Canopy-attention-YOLOv4-based immature/mature apple fruit detection on dense- foliage tree architectures for early crop load estimation. *Computers and Electronics in Agriculture*.

[36] Li X., Pan J., Xie F. (2021). Fast and accurate green pepper detection in complex backgrounds via an improved yolov4-tiny model. *Computers and Electronics in Agriculture*.

[37] Han K., Wang Y., Chen H. (2022). A survey on vision transformer. *IEEE Transactions on Pattern Analysis and Machine Intelligence*.

[38] Liu S., Qi L., Qin H., Shi J., Jia J. Path aggregation network for instance segmentation.

[39] Tan M., Pang R., Le Q. V. Efficientdet: scalable and efficient object detection.

[40] Qiao S., Chen L.-C., Yuille A. Detectors: detecting objects with recursive feature pyramid and switchable atrous convolution.

[41] Häni N., Roy P., Isler V. (2020). MinneApple: a benchmark dataset for apple detection and segmentation. *IEEE Robotics and Automation Letters*.

[42] Everingham M., Van Gool L., Williams C. K., Winn J., Zisserman A. (2010). The pascal visual object classes (voc) challenge. *International Journal of Computer Vision*.

[43] Russell B. C., Torralba A., Murphy K. P., Freeman W. T. (2008). LabelMe: a database and web-based tool for image annotation. *International Journal of Computer Vision*.

[44] Borji A. (2020). Empirical upper bound, error diagnosis and invariance analysis of modern object detectors. http://arxiv.org/abs/2004.02877.

[45] Zhou J., Jampani V., Pi Z., Liu Q., Yang M.-H. Decoupled dynamic filter networks.

[46] Guo J., Han K., Wang Y. Distilling object detectors via decoupled features.

[47] Mazumder P., Singh P., Namboodiri V. Cpwc: contextual point wise convolution for object recognition.

[48] Sun M., Song Z., Jiang X., Pan J., Pang Y. (2017). Learning pooling for convolutional neural network. *Neurocomputing*.

[49] Hu J., Shen L., Sun G. Squeeze-and-excitation networks.

[50] Davis J., Goadrich M. The relationship between precision-recall and roc curves.

[51] Rezatofighi H., Tsoi N., Gwak J., Sadeghian A., Reid I., Savarese S. Generalized intersection over union: a metric and a loss for bounding box regression.

[52] Paszke A., Gross S., Massa F. (2019). Pytorch: an imperative style, high-performance deep learning library. *Advances in Neural Information Processing Systems*.

[53] Chen K., Wang J., Pang J. (2019). Mmdetection: open mmlab detection toolbox and benchmark. http://arxiv.org/abs/1906.07155.

[54] Wang J., Chen K., Xu R., Liu Z., Loy C. C., Lin D. Carafe: content-aware reassembly of features.

[55] Wang J., Zhang W., Cao Y. Side-aware boundary localization for more precise object detection.

[56] Zhang H., Chang H., Ma B., Wang N., Chen X. Dynamic r-cnn: towards high quality object detection via dynamic training.

[57] Cao Y., Chen K., Loy C. C., Lin D. Prime sample attention in object detection.

[58] He K., Gkioxari G., Dollár P., Girshick R. Mask r-cnn.

[59] Rossi L., Karimi A., Prati A. A novel region of interest extraction layer for instance segmentation.

[60] Kuang H., Liu C., Chan L. L. H., Yan H. (2018). Multi-class fruit detection based on image region selection and improved object proposals. *Neurocomputing*.

[61] Zhao Z. Q., Zheng P., Xu S. T., Wu X. (2019). Object detection with deep learning: a review. *IEEE Transactions on Neural Networks and Learning Systems*.

[62] Cui L., Ma R., Lv P. (2018). Mdssd: multi-scale deconvolutional single shot detector for small objects. http://arxiv.org/abs/1805.07009.

